# A conceptual analysis of public opinion regarding genome research in Japan

**DOI:** 10.3389/fgene.2023.1170794

**Published:** 2023-11-28

**Authors:** Shibly Shahrier, Hristina Gaydarska, Kayo Takashima, Go Yoshizawa, Jusaku Minari

**Affiliations:** ^1^ Teesside University International Business School, Teesside University, Tees Valley, United Kingdom; ^2^ Center for iPS Cell Research and Application, Kyoto University, Kyoto, Kyoto, Japan; ^3^ Innovation System Research Center, Kwansei Gakuin University, Nishinomiya, Hyogo, Japan

**Keywords:** genome research, awareness, attitude, intention, cultural transmission, public communication

## Abstract

In the 20 years since the completion of the Human Genome Project, the gap between scientific development and public understanding of genome research has been widening. While genome research has been increasingly utilized for social and clinical purposes in a multifaceted manner, this has resulted in an increase in the potential risks associated with genomic data. In this context, our study aims to consider the nature of public perceptions of genome research, primarily by using as a case study the results of previous public surveys relevant to donations for social benefits in Japan. We explored certain types of awareness, attitude, and intention (A-A-I) in such surveys and discussed the resultant key findings through the cultural transmission framework. Reframing the public’s response toward genome research based on A-A-I analysis and behavioral science may contribute to developing more systematic communication approaches with the public. With a view to establishing such approaches, our perspective suggests some new insights to discuss the science–society gap in genome research internationally.

## 1 Introduction

Since the completion of the Human Genome Project, the gap between scientific development and the public’s understanding of genome research (GR) has been widening. This looming gap may relate to the complexity of genetic knowledge and divergent sets of views among scientific and societal stakeholders, emphasizing the importance of informed public engagement (Raz et al., 2022). Specifically, recurring challenges for sample donation exist, given that the handling of genomic data raises many concerns related to the public’s understanding of GR, such as privacy, consent, and ultimately trust (Borry et al., 2018). For the successful promotion of genomic medicine for the diagnosis and treatment of various diseases, dialogue with the public is essential, as scientific research is evaluated according not only to its scientific relevance but also to its value for society.

However, due to the increasing science–society gap related to GR, two significant aspects challenge its credibility. One concerns an individual *donating with eyes shut*, where respondents have been found to be willing to contribute despite a lack of trust (Raz and Hashiloni-Dolev, 2021). The other is when an individual *refuses to donate with eyes open* due to a lack of understanding and to mistrust. In the first case, participants may donate samples with little knowledge of terms such as *DNA* or *the human genome* and with ambivalent perceptions and trust regarding GR. In the second case, individuals may refuse to donate because they have insufficient knowledge and trust. Although a previous study showed that genetic literacy (GL) has improved over time (Little et al., 2022), regardless of whether the public *donates with eyes shut* or *refuses to donate with eyes open*, both scenarios might challenge the making of an informed decision. Therefore, for informed decisions, the public needs basic familiarity with GR and an understanding of the social benefits and risks related to it.

Many surveys have already been conducted to examine the public’s perceptions of how to contribute to GR in Western countries (Haga et al., 2013; Domaradzki and Pawlikowski, 2019; Little et al., 2022). However, public survey studies on people’s voluntary contributions to GR—a key promoter of this kind of research—are especially limited in non-Western countries. Given that GR development relies on the donations of diverse populations in international contexts (Atutornu et al., 2022), further considerations for underrepresented groups and non-Western countries is important. As Japan has participated in international projects such as the Human Genome Project and the International HapMap Project and national projects for GR and its clinical application (Minari et al., 2014; Minari et al., 2018), considering some of the characteristics obtained from Japanese public surveys can be beneficial to better represent the diversity of voices in international contexts.

This study explores the Japanese perspective toward voluntary donations based on the following three key aspects covered in previous GR public survey studies: awareness, attitude, and intention (A-A-I). In this study, we select past public surveys using a selection criterion—namely, questionnaire survey studies of voluntary donations for GR that contribute to future social benefits. Specifically, we refer to an individual’s decision to donate a sample biospecimen voluntarily. Past studies associated with healthy individuals who directly benefited from donations and with patients or participants in a particular project were excluded from the primary focus of this paper. In this study, we identify and analyze eight key Japanese papers related to GR (Ikeda, 2008; Ishiyama et al., 2008; Kobayashi and Satoh, 2009; Okita et al., 2018; Hishiyama et al., 2019; Middleton et al., 2020; Ri et al., 2022; Muto et al., 2023). To address the diversities of past Japanese studies on GR, this paper adopts a new theoretical approach—the conceptual framework of cultural transmission. This framework is used to systematically study the development of human behavior depending on transmission pathways, content, environment, and methods (Mesoudi and Whiten, 2008). Specifically, through the cultural transmission framework, we attempt to propose some new insights and suggestions to discuss the science–society gap and reflect on the findings from public surveys on GR. As GR becomes mainstream, it is hoped that our findings will aid effective communication between the many and diverse stakeholders involved.

## 2 Rationale

### 2.1 A-A-I elements

**FIGURE 1:**
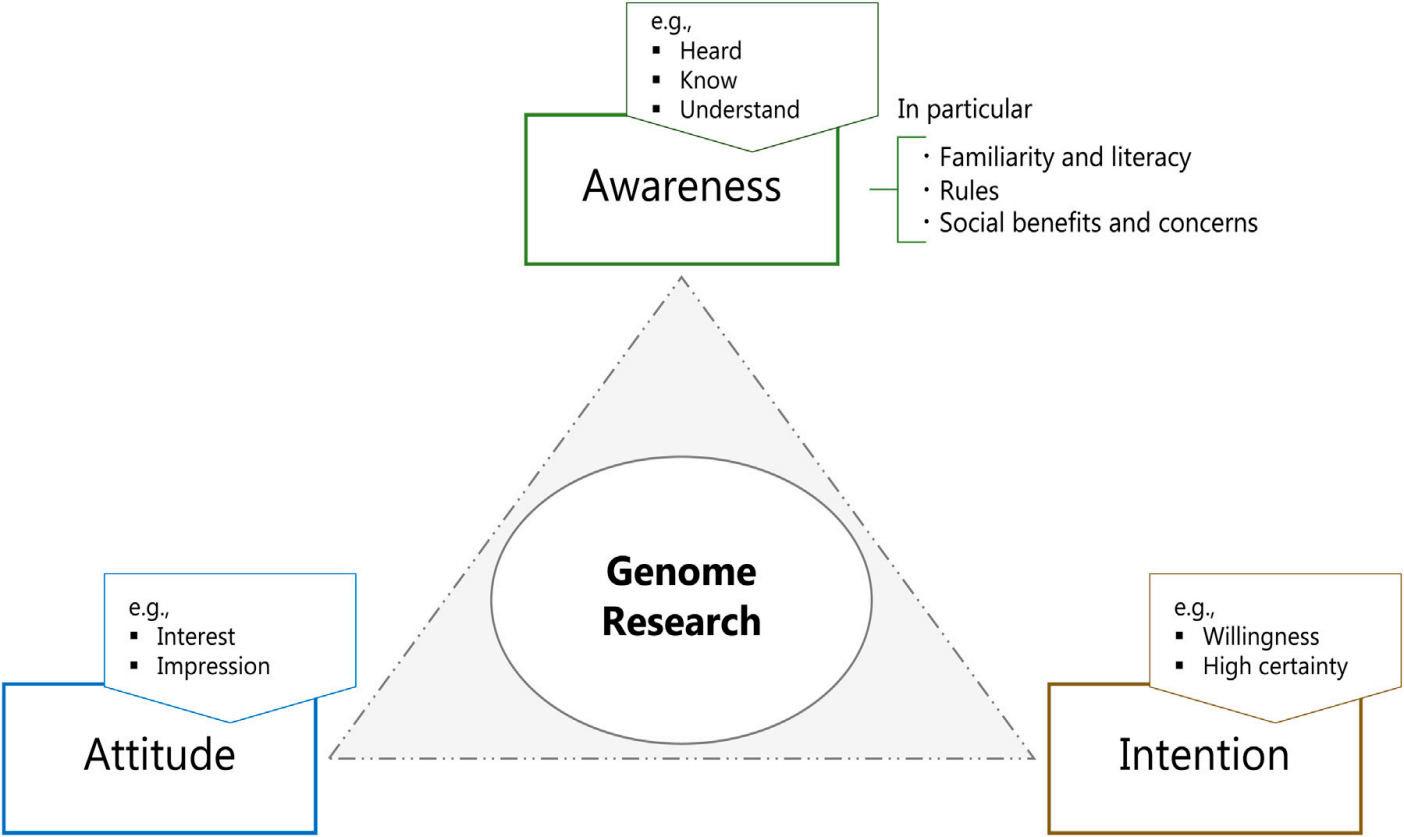
The A-A-I framework used to analyze and consider general public attitudes toward GR. For elements of awareness, the following three factors are explored: familiarity and literacy, rules, and social benefits and concerns.

This analysis focuses on A-A-I elements on GR, which could be predictors of actual voluntary contributions (Ajzen and Madden, 1986; Ajzen, 1991). Awareness refers to the perception of the scientific, regulatory, and social aspects of GR; attitude denotes general interest in and acceptance of GR; and intention is the specific interest and willingness to participate in GR with a high level of certainty (shown in Figure 1). Specific example terms of each theme used in the eight papers are delineated as follows:

1) *Awareness* can be defined as the perception of scientific and social aspects. In Japanese surveys, the notion has been described with terms such as *heard*, *know(ledge)*, *literacy*, *understand(ing)*, and *familiar(ity)*.
  We focus on the following three elements of awareness:
  (i) Genetic familiarity/literacy as *gene-related information, genetic information, *and *genomic studies*;
  (ii) Rules as *regulatory rules, ethics, guidelines, *and *law*;
  (iii) Future social benefits and concerns as *effective use, useful, privacy, genetic exceptionalism, *and *discrimination.*

2) *Attitude* denotes general interest in and acceptance of GR. Specifically, Japanese surveys use the terms *interest(ed)*, *concerns*, *preference*, *expect(-ation, -ed)*, *perception*, *wish to know*, *impression*, *necessary*, *opinion(s)*, and (*dis)agree.*
3) *Intention* concerns a specific interest in and willingness to participate in GR with a high level of certainty. Japanese surveys describe this notion as *willingness to participate/donate/undergo*.

In this study, we attempt to consider the findings of the A-A-I elements from eight previous Japanese studies, described in Section 3, and further discuss such perspectives (see Section 4) using the lens of cultural transmission theory.

### 2.2 Using cultural transmission theory to analyze A-A-I elements

An efficient way to explain the mechanism of behavior formation is through the framework of *cultural transmission and learning* (Darwin, 1859; Boyd and Richerson, 1985). Eminent researchers in various scientific fields, such as Charles Darwin, have indicated the importance of cultural transmission (Darwin, 1859; Boyd and Richerson, 1985), and a growing body of research has studied this process both theoretically and empirically (Moya et al., 2015). In human societies, cultural traits such as information, knowledge, beliefs, behaviors, and skills are transmitted as humans learn from other humans in various ways. This transmission process is called *cultural transmission/learning* (Mesoudi and Whiten, 2008). Unlike other social learning mechanisms in animal social groups, the cultural transmission mechanism prevails uniquely in human society, with people learning traits and taking on the perspective of the person from whom they are learning (Tomasello et al., 2005). As such, the process of cultural transmission is likely to contribute to awareness formation and impact people’s attitudes and intentions (Shahrier et al., 2016; Shahrier et al., 2017).

The formation of behaviors through cultural transmission depends on the following four key aspects: the pathways of transmission (who), the content of transmission (what), the environments of transmission (when), and the methods of transmission (how) (Cavalli-Sforza et al., 1982; Mesoudi and Whiten, 2008). In this study, we primarily focus on the pathways and content of transmission, since the findings in the selected papers are not closely related to the other two aspects.

Pathways of transmission refer to the source of cultural traits—that is, who is transmitting them. People transmit cultural traits among members of their generation and future generations through several pathways of cultural transmission. Representative examples of pathways of transmission are *vertical transmission* and *horizontal transmission*. While vertical transmission refers to transmission from parents to offspring (i.e., one-to-one or one-to-few transmissions), horizontal transmission refers to transmission among people in society through mass media, formal education, or famous individuals (Cavalli-Sforza et al., 1982; Mesoudi and Whiten, 2008). Vertical transmission can involve a higher level of perspective taking and trust in learning than horizontal transmission does because of the genetic and emotional closeness of parent–child transmission (Cavalli-Sforza et al., 1982; Tanskanen et al., 2021). Perspective taking refers to perceiving a situation from a position that is different from one’s actual position and adopting the perspective of another person (Herrera et al., 2018). For instance, if someone learns about GR because of the genetic conditions or diseases of their parents, they may think about GR from their parents’ viewpoint. This emotional process of learning is likely to trigger other-regarding preferences for patients beyond their parents who have genetic conditions. In contrast, horizontal transmission can reach a large number of people. If newspapers promote current developments in GR, a large number of people can come to know about them. In this regard, the transmission rate and range of vertical transmission may be much lower or narrower than those of horizontal transmission. Overall, the rate and range of transmission, the level of perspective taking in learning, and trust in the information vary depending on the pathways of transmission (Cavalli-Sforza et al., 1982; Boyd and Richerson, 1985; Tomasello et al., 2005).

Content of transmission refers to what traits (i.e., knowledge and information) are transferred to and copied by the receivers (Mesoudi and Whiten, 2008). These traits determine the kind of awareness that is growing among people and its impact on their behaviors. If people know more about the terminologies and developments in GR, their scientific knowledge will be enhanced. The transmission of information related to regulatory and social aspects will contribute more to the development of social and regulatory awareness of GR. However, knowledge and information are not passed linearly, since individuals make selective use of information—a situation called *content bias* (Boyd and Richerson, 1985; Mesoudi and Whiten, 2008). Several content biases are classified in cultural transmission as being related to the kind of information that is copied more easily–namely, i) payoff bias (i.e., a trait with a higher payoff being copied), ii) emotion bias (i.e., a trait provoking disgust being more likely to be copied than other traits), iii) threat bias (i.e., words related to potential threats and survival potentially being recalled better than other words), and iv) social bias (i.e., putting greater emphasis on a trait’s moral aspects than on its efficacy) (Mesoudi and Whiten, 2008; Stubbersfield, 2022). An example of biased transmission may be individuals exhibiting a more positive reaction toward GR in medical contexts than in other contexts. Compared to the nonmedical context, the probability of social transmission in the medical context might be proportionally higher, since it may relate to a higher perceived health-related threat. Thus, the content of transmissions may impact behaviors related to voluntary contributions to GR through awareness building, depending on what is transferred and copied by the receivers in the transmission process.

## 3 Awareness, attitude, and intention

In this section, we extract the Japanese perspective and tendency of A-A-I toward GR using eight Japanese papers compatible with our scope (summaries of these studies and of their A-A-I elements are given in Tables 1 and 2). The definitions of *awareness*, *attitude*, and *intention* are based on Section 2.1.

**TABLE 1:** Summary of the eight Japanese studies.

Year of Publication	Authors	Article title	Questionnaire type	Study year	Sample size
2008	Ikeda	The public’s attitudes towards the use of genetic information for medical purposes and its related factors in Japan	Questionnaires (each household to distribute in person and collect in person)	2004	151 individuals from the general population (Non-respondents or those who had “never heard the term genetic information” were excluded)
2008	Ishiyama et al.	Relationship between public attitudes toward genomic studies related to medicine and their level of genomic literacy in Japan	Postal questionnaires	2005	2171 individuals from the general population
2009	Kobayashi and Satoh	Public involvement in pharmacogenomics research: a national survey on public attitudes towards pharmacogenomics research and the willingness to donate DNA samples to a DNA bank in Japan	Online	2008	1103 individuals from the general population
2018	Okita et al.	Public attitudes in Japan toward participation in whole genome sequencing studies	Online	2015	2399 individuals from the general population
2019	Hishiyama et al.	The survey of public perception and general knowledge of genomic research and medicine in Japan conducted by the Japan Agency for Medical Research and Development	Online	2016	3000 individuals from the general population
2020	Middleton et al.	Global public perceptions of genomic data sharing: what shapes the willingness to donate DNA and health data?	Online	N/A	Japan = 4748
2022	Ri et al.	Expectations, concerns, and attitudes regarding whole-genome sequencing studies: a survey of cancer patients, families, and the public in Japan	Online	2021	10,077 ・Patients (having a history of cancer, CPs = 1204) ・The family members of patients with cancer (FMs = 5958) ・Adults from the general population (no personal/family history of cancer, GAs = 2915)
2023	Muto et al.	Is legislation to prevent genetic discrimination necessary in Japan? An overview of the current policies and public attitudes	Online	2017 and 2022	10,881 in 2017 4982 in 2022 (286 respondents who did not answer the questions on educational background in 2022 were excluded)

**TABLE 2:** Summary of the A-A-I elements in the eight Japanese studies.

Year of Publication	Authors	A-A-I*			Attitude	Intention
		Awareness	Attitude	Intention		
2008	Ikeda	×	×		Interest in the use of genetic information for medical research: 80% (extremely and somewhat interested), 20% (not very interested).	
2008	Ishiyama et al.	×	×		Are you interested in genomic studies related to medicine?: 71% (yes), 29% (other).	
2009	Kobayashi and Satoh	×	×	×	I think that identifying genomic markers associated with ADRs contributes to the safe use of drugs and therapy: 81% (agree), 2% (disagree).I think that a DNA-bank pooling DNAs of patients taking medications contributes to research on revealing the association between DNA and ADRs: 70% (agree), 2% (disagree).	I will donate my DNA for research when I take medications: 45% (I agree), 8% (I disagree).I will donate my DNA for research when I experience severe ADRs: 62% (I agree), 8% (I disagree).
2018	Okita et al.		×	×	Interest in and concerns about whole genome sequencing: 47% (interest; very much/moderately), 27% (interest; a little/not at all).	Willingness to participate in the whole genome sequencing study: 29% (very much/moderately), 37% (a little/not at all).
2019	Hishiyama et al.	×	×		Are you interested in research or medicine with respect to genetic information (genetic constitution)?: 46% (very much/moderate), 44% (not at all/a little).	
2020	Middleton et al.	×		×		Willingness to donate anonymous DNA and medical information to different recipient groups (doctor, non-profit, and for-profit researchers): ∼20–33% (Yes), ∼31–49% (No).
2022	Ri et al.	×		×		Participation in WGS studies: CPs 30%, FMs 27%, and GAs 18% (want to participate); CPs 20%, FMs 22%, and GAs 33% (don't want to participate).
2023	Muto et al.	×				

*Definitions in A-A-I are based on the definitions in Section 2.1.

### 3.1 Awareness

Concerning awareness, we explore awareness in the following three ways: genetic familiarity (GF) and GL, awareness of rules, and awareness of future social benefits and concerns.

#### 3.1.1 Genetic familiarity and genetic literacy

Six of the eight surveys included a certain aspect of GF (Middleton et al., 2020) and GL as components of awareness (Ikeda, 2008; Ishiyama et al., 2008; Hishiyama et al., 2019; Ri et al., 2022; Muto et al., 2023). Regarding GF, Middleton et al. (2020) reported that 88% of Japanese respondents answered *not familiar with DNA, genetics, or genomics*. This unfamiliarity with such words may come from the characters that the Japanese language uses, which involve four types of script—namely, hiragana and katakana (both phonograms), kanji (ideograms/logograms), and letters of the Latin alphabet for imported English expressions. In particular, the term *genomics* is translated phonetically using katakana as a loanword, but it is neither necessarily widely known in Japanese society nor translated into Japanese kanji, which is easy to understand for Japanese people because each letter has a meaning. Such Japanese expressions of technical terms can be associated with the nature of not only GF but also GL.

Except in two of the surveys, GL was explored subjectively as self-reported knowledge. Regarding the subjective genomic literacy in the surveys, different patterns of terms such as (*never*) *heard*, *know*, and *understand* existed. While the results of these surveys given in different contexts are not easy to compare, four main findings could still be identified. First, many respondents knew the terms *gene* and *DNA*. For example, Ishiyama et al. (2008) reported that 99% of the respondents knew or were aware of these terms in a survey conducted in 2005. Similarly, Hishiyama et al. (2019) recognized through a survey conducted about ten years later that these two terms were well known to, respectively, 93% and 91% of the participants in 2016. The term *gene* has already been translated into Japanese kanji as 遺伝子 and become widely known in Japanese society, while *DNA* has not been translated but has become commonly known (e.g., because of criminal investigations and paternity ascertainment). Nonetheless, the objective understanding of these terms considerably varied between respondents (Ishiyama et al., 2008). Relatedly, Ikeda (2008) reported that 81% of respondents who had heard of the term *genetic information* received genetic knowledge through mass media, while only 10% received genetic knowledge through public education. The second finding is that the Japanese people are not very aware of the term *genome*, which has been translated into katakana, compared to the terms *gene* and *DNA*. Ishiyama et al.’s (2008) survey reported that while 55% of the respondents were aware of the term *genome*, only 15% knew the meaning of that term, and 30% had never heard of it. The lower awareness of the term *genome* compared to that of the terms *gene* and *DNA* was observed in a public survey 10 years later (Hishiyama et al., 2019), indicating that this tendency has not significantly changed over time. Our third finding is that the term *genetic information* is relatively well known. Ikeda (2008) revealed that when *genomu jōhō* (ゲノム情報) and *idennshi jōhō* (遺伝子情報) were surveyed as *genetic information*, around 70% of respondents reported that they had heard of it. Still, another study by Hishiyama et al. (2019) reported that 45% of the respondents knew the term *genetic information*. Our fourth finding is that awareness of another term that has been increasingly incorporated into GR—*whole-genome sequencing* (*WGS*)—seemed to be relatively low among the respondents (29%, the sum of *know about it and have heard of it*), but recognition of the *WGS* study was relatively higher for the patient (43%) and family members of the patient (39%) category groups (Ri et al., 2022).

Our findings suggest that, to communicate with people, the selection and combination of words are important factors for the measurement of awareness. It is worth considering that awareness of technical terms can be largely influenced by their expressions and translations, particularly in countries with language systems that are distant from that of English. The proper selection and combination of terms in a public survey are applicable to other countries where heterogeneous comprehension of the basic genetic concepts exists (Luthuli et al., 2022). Also, the relationship between subjective and objective understanding of the terms should be carefully explored through further studies.

#### 3.1.2 Awareness of the rules

Three of the eight surveys (Ikeda, 2008; Hishiyama et al., 2019; Muto et al., 2023) touched on aspects of the rules of GR—namely, the existence of regulations, recognition of the content of the regulations, and perceptions of genomic information in the regulations.

Regarding the existence and recognition of regulations, Ikeda (2008) showed that the respondents were unaware of the existence of the *Ethical Guidelines for Human Genome/Gene Analysis Research* (43%), aware of the guidelines but not in detail (44%), or aware of them (13%). This study also reported that respondents were more aware of the existence of the *Act on the Protection of Personal Information* but not in detail (45%) or aware of the act (30%), compared with their awareness of these ethical guidelines. This discrepancy may be because, while the ethical guidelines are specific to GR, the *Act on the Protection of Personal Information* is associated with many activities in society that are not limited to GR, thereby creating public familiarity.

Nonetheless, about half of the respondents who were aware of the Japanese rules answered that these regulations were not substantively adequate (the sum of *don’t really think so* and *don’t think so at all*). It is necessary to address these responses through further research. The study also reported that, overall, 91% (the sum of *don’t really think so*, *don’t think so at all*, and *don’t know*) of the respondents perceived that these two regulations were not commonly known among the public at that time.

Related to this discussion, one paper reported that the necessity for legal regulations with penalties had increased from 2017 to 2022 (Muto et al., 2023) and that a new bill covering the prevention of genomic discrimination had recently been enacted. With regard to the handling of genetic information, Hishiyama et al. (2019) reported that 44% of respondents answered that GR should be implemented under stricter regulations than ordinary research, while 19% responded that GR should be implemented under the same regulations as ordinary research. The most common answer related to the necessity for stricter regulations was chosen by a high proportion of the participants who knew more than six genetic terms (60%), compared to a lower proportion of the participants who knew fewer than five terms (40%). Furthermore, the answer *I don’t know* (23%) was chosen by a higher proportion of the participants who knew fewer than five terms (72% compared to 28%), indicating that people with less knowledge had difficulties responding.

#### 3.1.3 Awareness of social benefits and concerns

Five out of eight surveys explored awareness of social benefits and concerns (Ikeda, 2008; Kobayashi and Satoh, 2009; Hishiyama et al., 2019; Ri et al., 2022; Muto et al., 2023). Regarding social benefits, in four papers, 38%–85% of the Japanese public perceived the usefulness of GR positively. Muto et al. (2023) showed that regarding the social benefits categories of GR, such as diagnostics, treatment, and prevention of diseases, in 2017, more than 90% responded to the three options of *agree* (15%–18%), *tend to agree* (41%–48%), and *cannot say* (28%–35%) rather than *tend to disagree* or *disagree*. Similar results, obtained in 2022, showed that the percentage of respondents who expressed *tend to agree* and *agree* slightly increased across all categories over time (except for the reduction of unnecessary medical expenses). According to Ri et al. (2022), more than 85% of respondents answered with the three options *agree* (7%–12%), *somewhat agree* (31%–44%), and *neither agree nor disagree* (37%–51%) rather than *somewhat disagree* or *disagree* for social awareness categories. In this study, while more than half of the respondents were aware of social benefits for most categories, a relatively lower number perceived that benefits were obtained in reducing the health-care costs (38%, the sum of *somewhat agree* and *agree*). In addition, Ri et al. (2022) showed that patients with cancer and the family members of patients with cancer were more likely to perceive such benefits more strongly than general adults (e.g., in the category of cancer diagnosis: patients, 75%; family members, 71%; and general adults, 54%). As another example, Ikeda (2008) demonstrated that most participants responded more to the two options *extremely useful* (48%) and *somewhat useful* (36%) than to *don’t know*, *not very useful*, and *not useful at all* when asked about perceptions of one’s possibility for having future disorders. This study also focused on responses from those who had heard the term *genetic information* and found, in contrast to Muto et al. (2023) and Ri et al. (2022), that higher awareness of social benefits (i.e., *extremely useful* [48%]) seemed to be obtained from people more knowledgeable about GL.

These results show at least three key findings about the social benefits of GR. First, at least about half of the respondents were aware of the usefulness of GR in general, with about 10%–15% and 40% of them clearly expressing *agree* and *tend to agree* or *somewhat agree,* respectively. In this regard, relatively less awareness could be seen regarding the reduction of medical costs. Second, many more respondents expressed their awareness of the social benefits of GR. Relatedly, among the respondents who had some degree of GL, more people showed high awareness (*extremely useful*). Last, both patients with cancer and the family members of patients with cancer perceived more of these benefits than did general adults.

Similarly, 8%–70% of the Japanese public perceived concerns related to GR in four papers. Muto et al. (2023) showed that regarding social concern categories tied to GR—such as handling of genetic information in medical institutions, administrative agencies, and genomic discrimination—in 2017, more than 85% of the participants responded *agree* (8%–16%), *tend to agree* (29%–33%), and *cannot say* (44%–48%), rather than *tend to disagree* and *disagree*. Similar results were obtained in 2022; over time, while the percentage of respondents who selected *tend to agree* and *agree* slightly decreased across all categories, the percentage of respondents who answered *cannot say* slightly increased across all categories. According to another study, by Ri et al. (2022), the general population mostly responded *agree* (8%–19%), *somewhat agree* (31%–37%), and *neither agree nor disagree* (40%–50%), rather than *somewhat disagree* and *disagree*, for awareness of concern categories. The authors of this study also reported that cancer patients and individuals with family members who were cancer patients were more likely to perceive such concerns more strongly than general adults—slightly higher for the family members than for the patients in most categories (except for inequalities in access to health-care). In particular, 63% of the family members (the sum of *somewhat agree* and *agree*) expressed concerns regarding the privacy of genetic information, which was even higher than the concern of the patients (61%) and that of general adults (53%). According to Hishiyama et al. (2019), the respondents paid more attention to the reliability of genetic information (48%), strict management systems (37%), the purposes of research (32%), and avoiding discrimination (22%), while they paid less attention to job category to explain genetic information (8%) and to experts’ attitudes toward returning individual genetic research results (8%). Last, Kobayashi and Satoh (2009) clarified that the respondents had greater concerns about the confidentiality of personal information (70%) and the handling and publishing of research results (52%) than about the use of their DNA for research purposes (26%) and the possibility of a specific genetic condition being revealed (24%).

These results show at least five key findings regarding concerns about GR. First, about 40%–50% of the respondents were aware of various concerns about GR, with about 10%–15% and 30%–35% of the respondents seeming to clearly express *agree* and *tend to agree* or *somewhat agree*, respectively. Second, about 45% of the respondents answered *cannot say* or *neither agree nor disagree* for various concerns. Third, most respondents expressed their concerns about confidentiality, especially in the case of GR within specific contexts. Fourth, the respondents may have tended to consider issues related to the handling of genetic information rather than to the behavior of professionals. Last, cancer patients and cancer patients’ family members perceived such concerns more than did general adults.

### 3.2 Attitude

Attitude can be broadly defined as a complex combination of values, beliefs, and motivations (Pickens, 2005). In five out of the eight studies, a significant percentage of the respondents had a positive attitude toward GR promotion, with some degree of difference in attitudes (Ikeda, 2008; Ishiyama et al., 2008; Kobayashi and Satoh, 2009; Okita et al., 2018; Hishiyama et al., 2019). Respondents showed a high interest in GR in Ikeda (2008) (80%, the sum of *extremely* and *somewhat interested*) and Ishiyama et al. (2008) (71%). In this regard, attitudes toward the medical context were more positive than toward the nonmedical context (Ishiyama et al., 2012). Similarly, 47% (Okita et al., 2018) and 46% (the sum of *very much* and *moderate*) (Hishiyama et al., 2019) of respondents exhibited an interest in the *WGS* study and GR, respectively. Notably, in the study by Kobayashi and Satoh (2009), the attitudes toward pharmacogenomics research and DNA banks were relatively higher, at 81% and 70%, respectively, indicating that specific contexts could increase positive attitudes.

### 3.3 Intention

Intention is defined as a specific interest in and willingness to participate in GR. Intention toward GR was explored in four out of the eight studies (Kobayashi and Satoh, 2009; Okita et al., 2018; Middleton et al., 2020; Ri et al., 2022). Except for the study by Kobayashi and Satoh (2009), the range of intention obtained from the studies was 18%–33%. Okita et al. (2018) and Ri et al. (2022) focused on the willingness to participate in the *WGS* study and Middleton et al. (2020) examined the willingness to donate DNA samples for multiple users, including doctors and nonprofit/for-profit organizations. The willingness to participate in GR was 29% in the study by Okita et al. (2018), 20%–33% in the study by Middleton et al. (2020), and 18% in the study by Ri et al. (2022). In addition, Ri et al. (2022) reported that general adults tended to be less willing to participate in the *WGS* study (18%) compared to patients (30%) and patients’ families (27%). However, such willingness may increase when the respondent can comprehend the specific research aim, target, or content. Kobayashi and Satoh (2009) showed respondents’ willingness to donate DNA samples for a specific genetic condition using two hypothetical situations—namely, when taking medications (45%) and when experiencing severe adverse drug reactions (62%). This result indicates that without receiving individual benefits from GR—such as, the return of genome information—the willingness to donate may differ depending on the content of the research or whether the research purpose is clarified.

### 3.4 Relationships between A-A-I elements

The relationships between the A-A-I elements were explored in five out of the eight studies (Ikeda, 2008; Ishiyama et al., 2008; Kobayashi and Satoh, 2009; Hishiyama et al., 2019; Middleton et al., 2020). In our analysis, we identified the following three relationships: between awareness (GL and social benefits) and attitude, awareness (GF) and intention, and attitude and intention. Regarding GL and attitude, Ishiyama et al.,’s 2008 results from a regression analysis showed a positive relationship between the score of GL and approval of the promotion of GR. In other words, respondents with a higher GL were more likely to have a positive attitude toward GR. This relationship is supported by a recent study conducted by Hishiyama et al. (2019) that showed a positive relationship between GL and attitude. Regarding social benefits and attitude, in the study by Ikeda (2008), the awareness of GR’s usefulness for *making effective use of medicine* showed a positive relationship with the attitude toward GR, and the awareness of GR’s usefulness for *determining disorders to which one may be susceptible in the future* showed a negative relationship with the attitude toward GR. In particular, the latter implies that determining potential susceptible disorders might not necessarily be a positive influential factor in attitude toward GR. Regarding GF and intention, Middleton et al. (2020) found that people having higher GF or having had a personal experience related to GR were more likely to be willing to participate in GR, indicating that there is a positive relationship between GF and intention. Regarding attitude and intention, Kobayashi and Satoh (2009) revealed that a positive attitude toward a DNA bank was significantly associated with an increased willingness to donate, indicating that there is a relationship between attitude and intention. From the results of these previous surveys, it can be said that while positive relationships exist between A-A-I elements, both positive and negative relationships between awareness of social benefits and attitude have been observed.

During the analysis of A-A-I relationships, an intrasectional relationship of awareness was found in two papers (Hishiyama et al., 2019; Muto et al., 2023). Regarding the relationship between GL and awareness of rules, Hishiyama et al. (2019) demonstrated that knowledgeable participants, compared to less knowledgeable ones, perceived the necessity for stricter regulation of GR than of other, ordinary types of research. Similarly, regarding the relationship between GL and awareness of concerns, Hishiyama et al. (2019) suggested that knowledgeable participants, compared to less knowledgeable ones, regarded the strict management system of genetic information to be a key factor in the handling of genetic information, more than other elements. Regarding the relationship between GL and rules and between rules and social benefits, Muto et al. (2023) reported two findings—namely that both knowledgeable people (having subjective and objective knowledge of GL) and people who perceived both benefits and concerns regarding the use of genetic information were more aware of the necessity for penalties in cases of the misuse of genetic information and discrimination based on it. These two papers suggested that people with higher GL, and those who perceived social benefits and concerns regarding GR, could favor a stricter management of genetic information and the need for penalties.

In summary, previous Japanese studies focused more on the relationship between awareness and attitude as well as the intrasectional relationship of awareness. However, there is less evidence for relationships among the other interrelationships, such as awareness and intention and attitude and intention. Particularly interesting studies could explore the relationship between the awareness of social benefits and risks and intention and between the awareness of rules and intention.

## 4 Discussion

### 4.1 Conceptual analysis through cultural transmission

To further systematize the summary of A-A-I variations (Section 3), we used the cultural transmission framework. The key finding from this investigation involves the less explored relationships among A-A-I elements. Representative examples include the relationships between i) awareness and intention and ii) attitude and intention. By adopting the cultural transmission theory, some key aspects of these human behaviors regarding GR can be systematically understood. Specifically, we obtained two main findings using the A-A-I framework, related to *who* transmits the information (*pathways of transmission*) and *what* information is transmitted (*content of transmission*).

First, previous studies reported that most information on GR is mainly obtained through mass media (Ikeda, 2008; Ishiyama et al., 2012). In particular, Ishiyama et al. (2012) reported that about half of people were likely to obtain information on GR via newspapers and books as well as through mass media (television, radio). Conversely, family or friends were reported as a source of information by only 5% of the respondents. Given that these results were obtained more than 10 years ago (Ishiyama et al., 2012), it is likely that the internet has become another possible major transmission pathway. Theoretically, these results indicate that the previous pathways of transmission of GR were primarily horizontal, not vertical. This indicates that the pathways possibly contributed to a faster diffusion of knowledge and awareness building but impacted intentions less. However, since vertical transmission involves greater perspective taking relative to horizontal transmission, it may trigger other preferences, such as sympathy, compared to horizontal transmission. Indeed, the intention to participate in GR seems to be enhanced in cases that involve personal experience (Middleton et al., 2020; Ri et al., 2022), possibly due to higher perspective taking. Now that GR has been increasingly utilized in society, we must consider the nature of vertical and horizontal transmission, and their combinations, for public communication.

Regarding the content of transmission, Ishiyama et al. (2012) demonstrated that people, in general, can show a more positive attitude toward the medical setting than the nonmedical setting. One of the main reasons for this attitude can be explained by a possible threat bias (Mesoudi and Whiten, 2008; Stubbersfield, 2022)—that is, people may consider diseases the greatest threat to their lives. Our analysis indicates that while people are aware of basic genetic terms, social benefits, and concerns, they may not be familiar with some of the specific technical terms and rules of GR. In particular, theoretically, the awareness of social benefits can correspond to the payoff bias, since people are likely to support GR based on its potential social benefits. In this regard, when the information related to social benefits is delivered, payoff bias should be carefully managed in future public communication related to GR. On the other hand, the awareness of concerns and rules are mainly related to social bias involving the moral aspects of GR (e.g., genetic discrimination protection), as suggested by the theory of cultural transmission (Mesoudi and Whiten, 2008; Stubbersfield, 2022). To deliver information regarding concerns and rules, the impact of social bias must be seriously considered as well. These perspectives can play a key role in complementing and balancing such content of transmission.

### 4.2 Suggestions based on cultural transmission

For further studies and initiatives to develop more balanced public communication about GR, we suggest the following five approaches using cultural transmission: 1) practices of inverse vertical transmission (pathways of transmission); 2) delivery of complemented content through horizontal transmission (pathway and content of transmission); 3) codevelopment of GR and public values using interactive communication (pathway and content of transmission); 4) exploration of two other aspects of cultural transmission theory; and 5) attention to consistency, comparability, and multiscale questions on surveys.

#### 4.2.1 Practices of inverse vertical transmission

In Japan, it is likely that the general public mainly obtains information about GR through mass media (Ikeda, 2008; Ishiyama et al., 2012) and that the social environment rather than school education plays a key role in enhancing GL (Hishiyama et al., 2019). Regarding pathways of transmission, since perspective taking in vertical transmission triggers other-regarding preferences and horizontal transmission is effective for the faster diffusion of knowledge, a combination of them may be useful for public communication on GR. Considering pathways of transmission, encouraging informal child–parent inverse vertical transmission may be useful for developing further public communication on GR. In this pathway, school-aged children would learn about GR through formal education (horizontal transmission) and be encouraged to transfer that knowledge and information to their parents or provide triggers for them to learn about GR (inverse vertical transmission), possibly inducing the interactive development of children’s and parents’ behaviors related to GR. This approach could also be effective for addressing a fundamental issue, which is that it often takes a long time for research participants to receive direct, individual benefits.

#### 4.2.2 Delivery of complemented content through horizontal pathways

Regarding the content of transmission delivered through horizontal pathways, more complemented and balanced information should be delivered in Japan. At least, past Japanese public surveys on GR suggested the necessity for more awareness of several specific elements—namely, some of the technical terms (e.g., genomes), rules, benefits regarding medical cost reduction, and concerns. In particular, while many respondents were concerned with the handling of genetic information and confidentiality, a considerable number hesitated to provide decisive responses to their choices on the awareness of concerns (*cannot say* or *neither agree nor disagree*). A possible reason for this result may come from the possibility that the general public obtains less information regarding concerns about GR through main sources (e.g., mass media). In addition, an international study reported inaccurate perceptions of the respondents about GR, such as cloning and bioweapons from biobanking donations (Ahram et al., 2022). These findings suggest that social benefits (e.g., benefits to a target community), concerns, and rules (e.g., protections against genetic discrimination) must be fairly delivered to the public using social and mass media, given to the payoff and social biases. Moreover, through successful and famous individuals’ involvement in the delivery of information about GR (covering not only scientific aspects but also regulatory and social ones), the general public’s reaction to GR could be influenced by the perspectives of these individuals. This approach can be supported by a cultural transmission study that revealed that people emulate the behaviors of successful and prestigious individuals (Henrich and McElreath, 2003).

#### 4.2.3 Codevelopment of GR and public values using interactive communication

While the pathways and content of transmission suggested above can contribute to developing communications from core stakeholders such as policy officers, experts, and mass media to the general public, interactive communications from the general public to the stakeholders are also imperative for the codevelopment of GR and the public values. Given that one major characteristic of GR is the necessity for voluntary donations from the general population, key stakeholders in GR must ensure information transmission from the general public to GR practitioners through active and continuous dialogue. This interactive form of communication can create a social system of learning characterized by the efficient use of transmission pathways and content. In this regard, the language and content used in GR transmission should be carefully selected and communicated, since different terminologies can induce different perceptions and transmission biases (Ishiyma et al., 2008; Mesoudi and Whiten, 2008; Hishiyama et al., 2019). On the basis of such GR environments, if GR practitioners inform research participants of relevant evidence-based research outputs and, if feasible and necessary, of incidental clinical findings (which significantly concern diagnosis and treatment for participant health conditions), such initiatives may motivate the general public to seriously address the nature of GR. To consider international comparisons of GR, more careful attention to diverse groups and cultures is necessary. Sheikh and Hoeyer (2018) suggested that trusting stakeholders in communication is conditional on culture. To address cultural and other variations in the perception of GR, including mistrust, it is crucial to focus on the importance of shared values and community engagement. In general, where there is less direct interpersonal connection between the public and other stakeholders in GR, shared values can play a key role in enhancing trust (Passmore et al., 2019). Such shared values can be produced through institutes and community engagement (Buseh et al., 2013; Cohn et al., 2015; Shahrier et al., 2016).

#### 4.2.4 Exploration of two other aspects of cultural transmission theory

While our analysis paid significant attention to the pathways and content of transmission, the other two elements of cultural transmission—namely, environments and methods of transmission—can also be useful. Environments of transmission, referring to whether the content and environment of transmission change (i.e., a static or dynamic environment), are likely to affect the receivers’ level of trust in the information received and the frequency of learning (McElreath et al., 2005; Mesoudi and Whiten, 2008). For instance, receivers’ level of trust in the information and frequency of learning may be higher in a relatively static, rather than a dynamic, environment. On the other hand, methods of transmission, intended as the means of transmitting or learning (e.g., imitation, written or verbal language, identifying how traits are learned), may be relevant to the longevity of the information received in individuals’ memories. In this regard, Luthuli et al. (2022) suggested using video graphics and imagery-based storytelling as well as slowly explaining and involving friends or relatives to transfer a large volume of information to the participants. Thus, the environment of transmission can largely affect trust in information for awareness building, while the longevity of the information received regarding GR in human memory can partly depend on the method of transmission. Specifically, future public surveys on GR should address the four elements of cultural transmission in a well-balanced manner.

#### 4.2.5 Attention to consistency, comparability, and multiscale questions on surveys

Effective survey models to systematically obtain and compare public opinions on GR should be developed. The models of cultural learning are widely used to study human behavior, and they can be applied to study people’s perceptions of cooperating with GR. In this regard, our A-A-I framework could be useful as an attempt to explore such considerations of GR. An important implication of cultural transmission is that vertical transmission can promote prosociality for future generations (Shahrier et al., 2017). Specifically, since there is the potential to effectively communicate with the public through inverse vertical transmission, and thereby trigger prosociality for future generations in GR, further studies should examine this possibility. Last, most of the previous studies, which assessed people’s attitudes and intentions toward GR using a single question (e.g., self-reported knowledge), might be contextually insufficient and lack internal and external reliability and validity (e.g., objective vs. self-reported knowledge). For this challenge, multi-item scales for attitude and intention—involving a pre-examination of reliability and validity—may contribute to addressing such challenges related to the questions. Still, questions need to be compatible with the respondents’ level of comprehension. Survey design can be particularly challenging in countries where genomic literacy is low, and extensive and incomprehensible survey questions could divert respondent perception.

## 5 Conclusion

Despite the great effort of public surveys to acknowledge and address challenges associated with public perception, there is an imbalance between the understanding of the medical application and the social consequences of GR. Our conceptual analysis suggests ways to decrease the gap between science and society and highlights the need for a more unified approach toward public surveys. A systematic use of cultural learning would increase GF and GL while also bridging the gap between genomic hype, hopes, and awareness of the social implications surrounding GR. Even if public awareness can be acknowledged and potentially shaped, accurately measuring and predicting attitudes may be more difficult. In contrast to awareness, attitude includes personal, family, cultural, and ethical values, beliefs, and norms. As a multidimensional category, attitude is dynamic and changes with time; therefore, it is not easy to verify. However, investigating Japanese surveys and other international perspectives is a critical first step in learning from one another in the effort to effectively communicate with the public and build a resilient and inclusive environment for the promotion of science and technology. Internationally, initiatives to address underrepresentation in GR through community engagement and an understanding of the diversity of voices in international contexts are growing (Lemke et al., 2022). Our study of Japanese public surveys can contribute to the efforts of other countries where GR is not as common, and it can help to develop further understanding of the public’s perspectives on GR. Ultimately, we hope that our analysis will be useful for future studies by providing further insights into the nature of public surveys.

## Data Availability

The original contributions presented in the study are included in the article/supplementary material; further inquiries can be directed to the corresponding authors.
